# Mindfulness Practice and Burnout: Evidence From Chinese Social Workers

**DOI:** 10.3389/fpsyg.2022.821899

**Published:** 2022-02-24

**Authors:** Bin Tu, Chienchung Huang, Sophie Sitar, Meifen Yang

**Affiliations:** ^1^School of Public Administration, Guangdong University of Foreign Studies, Guangzhou, China; ^2^School of Social Work, Rutgers, The State University of New Jersey, New Brunswick, NJ, United States; ^3^Rutgers Law School, Rutgers, The State University of New Jersey, Newark, NJ, United States

**Keywords:** mindfulness practice, burnout, work, social worker, job demand

## Abstract

Over the span of nearly 10 years, the social work labor force grew from 0.2 million to approximately 1.2 million in China. Despite these increases, studies have shown social workers in China are also experiencing equally high burnout rates. For this analysis, we collected data from 537 social workers based in Guangzhou, China. We used the job demands and resources (JD-R) theory, to examine the relations between JD-R and burnout and whether mindfulness practice (MP) could reduce any such burnout. Our results suggest JD-R affects social workers’ burnout through both health and motivation impairment. High job demands (JD) were linked to high burnout while high job resources (JR) were linked to a reduction in burnout. Formal (Beta = −0.08) and informal (Beta = −0.19) MP were associated with low burnout amongst social workers. The significant interaction between JD and MP also suggests that MP can reduce burnout for social workers with high JD. The findings call for using MP to be used to shield social workers from the effects of increasing JD and to prevent an increase of burnout amongst Chinese social workers.

## Introduction

Since the 1980s, China’s economy has been rapidly developing, and so has the need for social workers to address rising social welfare needs. Therefore, there have been enormous expansions to China’s social work academic programs within the last 40 years. From 1989 to 2018, social work undergraduate, master’s, and Ph.D. programs have risen from tens of annual graduating students to over 40,000 annual social work graduates (Tang and Li, 2021). In tandem to rising social work academic institutions, the national Chinese social work labor force also drastically increased from 0.2 million in 2010, to 1.2 million in 2018 (Li et al., 2012; Chan et al., 2020). These statistics highlight just how quickly the field of social work has expanded throughout China, in a relatively short period of time. The future of social work in China, however, has become threatened by high burnout and turnover rates amongst social workers across the country (Lin and Lan, 2014; Xu et al., 2016; Jiang et al., 2019; Su et al., 2020; Tang and Li, 2021).

Internationally, social workers, like other human service workers, experience significant burnout (Lizano, 2015; Sánchez-Moreno et al., 2015). This is problematic because cross-cultural research shows industries with elevated burnout rates generally also have equally high turnover rates (Abu-Bader, 2000; Mor Barak et al., 2001; Lloyd et al., 2002; Evans et al., 2006; Kim and Stoner, 2008; Luo and Lei, 2021). For example, the turnover rates amongst social workers were around 23% in the United Kingdom (Hussein et al., 2016) and above 30% in the United States (National Child Welfare Workforce Institute, 2011; Boyas et al., 2015). Chinese social workers similarly experience these same elevated rates of burnout and thus subsequent high turnover (Lin and Lan, 2014; Du, 2015; Zhu, 2015; Jiang and Wang, 2016; Xu et al., 2016; Wang et al., 2019). Since the late 2000s, Guangzhou and Shenzhen social workers have been quitting their jobs in increasing numbers and turnover rates have been on the rise. For example, in 2014, 25% of Guangzhou social workers quit their jobs, (Zhu, 2015), and by 2015, approximately 18% of Shenzhen social workers had also left their positions (Du, 2015). In 2013, the Shenzhen social worker turnover rate was over 22% (Du, 2015). Additionally, using the Maslach Burnout Inventory (MBI) to measure burnout amongst social workers in China, various studies have found the average burnout was 53.9 out of 102 in Chengdu (Xie et al., 2021), and was 56.6 in Guangzhou (Wang et al., 2019). These two Chinese cities’ burnout levels appear to fall in the middle of international range. Comparatively, Israel ranges from 47.1 (Hamama, 2012) to 46.7 (Tartakovsky and Walsh, 2016); the United States is approximately 53.7 (Himle et al., 1986); Norway is approximately 54.0 (Himle et al., 1986); Spain is approximately 58.1 (Gómez-García et al., 2020); and Russia is approximately 62.6 (Zaitseva and Krikunov, 2021).

Increased burnout and turnover rates will generally impose negative consequences on not only the field of social work, but also on its labor force and its client populations (Social Work Policy Institute, 2010; Child Welfare Information Gateway, 2016; Casey Family Programs, 2017). For example, social workers who experience burnout may be unable to maintain quality client services, which can create harm, especially to their more vulnerable clients (Hamama, 2012; Casey Family Programs, 2017; Xie et al., 2021). Additionally, high turnover rates may force agencies to become understaffed and thus social workers who remain in the labor force are working with increased job demands and workloads (Yürür and Sarikaya, 2012; Child Welfare Information Gateway, 2016). Moreover, when caseworkers leave their jobs, agencies incur high costs to replace the staff member. These costs can be as high as 200 percent of the exiting employee’s annual salary and can include direct costs related to worker separation, hiring and training new staff, costs for administrative work to document the employee’s termination, costs for emotional exhaustion, and cost of recurrence of targeted behavior problems from clients (CPS Human Resource Services, 2006; Casey Family Programs, 2017).

This level of work stress and pressure can then, again, lead to further burnout amongst workers, which exacerbates the turnover cycle further. It is critical scholars investigate the contributing factors of burnout and mitigate these effects to better reform the social work industry in China. Without any such research or change, Chinese social workers maybe become unable to sustain long-term quality services.

According to JD-R theory, JD and JR are two separate categories of working conditions that can affect social worker burnout and other job-related issues (Bakker and Demerouti, 2007, 2018). First, JD refer to the various work conditions (i.e., physical, social, or organizational) that require or impose a continuous physical or mental effort. Often, this continual effort imposes a physiological cost, such as exhaustion and fatigue onto the worker. Second, JR refer to the aspects of the job (i.e., psychological, social, or organizational) that assist social workers to achieve their work goals, alleviate the psychological penalties of increased job demands, and/or encourage individual growth and progress, regardless of increasing workloads. JD and JR affect burnout and psychological well-being through health-impairment and motivation-driven processes (Bakker and Demerouti, 2007, 2018). In the former, JD cause workers to experience a gradual energy depletion, leading to psychological distress and ultimately burnout. In the latter, without JR, social work employees are more likely to fail at fulfilling their work roles or responsibilities, which, can lead to frustration, withdrawal, and, thus, distress and burnout.

Job burnout is a psychological effect of negative work-stress. Symptoms often include emotional fatigue, depersonalization, and a diminished sense of individual accomplishment, and are particularly heightened in challenging work conditions (Maslach et al., 1996). Burnout has been known to affect almost all professionals throughout the human service industry (Aiken et al., 2002; Hakanen et al., 2006), including social workers (Travis et al., 2015). JD-R has also been found to contribute to burnout amongst social workers and other human service professionals (Tang et al., 2017; Su et al., 2020). Numerous studies of both seasoned and amateur Chinese social workers (Tang et al., 2017; Tang and Li, 2021), demonstrate positive relationships between JD, burnout, and turnover, as well as negative relationships between JR and burnout (Su et al., 2020; Luo and Lei, 2021).

Hypothesis 1: JD are positively associated with burnout, while JR are negatively associated with burnout.

Mindfulness is a state of consciousness during which an individual actively engages in purposeful awareness and attention to the present moment, while maintaining non-judgmental reactions to their observations (Hanh, 1976; Kabat-Zinn, 1990, 2003; Baer et al., 2006). Studies on mindfulness and its association with a myriad of positive effects have proliferated in recent decades, including social and emotional competence, health, and well-being (Schonert-Reichl and Lawlor, 2010; Branstrom et al., 2011; Zoogman et al., 2014; Klingbeil et al., 2017; Song et al., 2021). Past research also suggests that mindfulness can also help regulate stress reactions (e.g., Roeser et al., 2013; Taylor and Millear, 2016). Findings from a study conducted by Hülsheger et al. (2012) found that mindfulness was negatively associated with emotional exhaustion and positively associated with job satisfaction in a sample of 219 working adults. Similarly, in a sample of 415 nurses, mindfulness buffered the relation between JD and psychological stress (Grover et al., 2017). Thus, mindfulness may act as a personal resource that reduces burnout and work stress in the JD-R model (Taylor and Millear, 2016; Grover et al., 2017; Guidetti et al., 2019). For example, mindfulness can be utilized to help an individual remain calm and objective when faced with thoughts or feelings that elicit emotional responses. Or it can help an individual’s ability to stay present and aware in the moment while ignoring or sidestepping potential distractions within the workspace. By attending to the present moment and achieving non-judgmental awareness of stressors brought on by various JD, employees can potentially utilize mindfulness to mitigate the negative effects of JD on stress and burnout (Greeno et al., 2018; Smith et al., 2019).

There is growing evidence that practicing mindfulness can reduce stress and improve mental well-being, both of which were associated with low burnout (Brown and Ryan, 2003; Taren et al., 2013; Hanley et al., 2015; Yang et al., 2018; Birtwell et al., 2019; Karr, 2019; Shankland et al., 2020; Song et al., 2021). Practicing mindfulness is about being fully aware of what is happening in the present moment, acknowledging one’s thoughts, feelings, and body sensations with compassion and devoid of all judgment.

Mindfulness can be practiced formally or informally. Formal practice, such as meditation, is a set of techniques that are intended to encourage a heightened state of awareness and focused attention (Hanh, 1976; Kabat-Zinn, 2003; Mason et al., 2019). An informal practice involves using mindful awareness in daily activities, such as eating, walking, dishwashing, or exercising (Hanley et al., 2015; Birtwell et al., 2019; Shankland et al., 2020). Recent studies have shown that both formal and informal MP were associated with low stress and increased mental health (Hanley et al., 2015; Yang et al., 2018; Birtwell et al., 2019; Shankland et al., 2020). For example, Birtwell et al. (2019) collected data from 218 adults who were practicing mindfulness and found that both formal and informal practice had significant positive effects on mental well-being. Frequency of informal practice appeared to have a slightly higher correlation with well-being (*r* = 0.33, *p* < 0.001) than frequency of formal practice did (*r* = 0.30, *p* < 0.001).

Hypothesis 2: Practicing mindfulness, both formally and informally, is negatively associated with burnout.

Hypothesis 3: The effects of JD on burnout are lower for individuals with high MP.

In short, the JD-R theory has been widely tested and numerous studies have affirmed its reliability to examine burnout, job stress, work engagement, and health (Schaufeli et al., 2009; Schaufeli and Taris, 2014; Lonska et al., 2021; Robina-Ramírez et al., 2021; Tang and Vandenberghe, 2021; Tesi, 2021). However, few have focused on the well-being of Chinese social workers. Further, little information is available on whether practicing mindfulness would reduce burnout of social workers in China. Thus, in this study, we employed the JD-R theory to examine the effects JD-R had on burnout and how MP could mitigate burnout amongst a sample of Chinese social workers. The study’s findings can advance the understanding of how JD-R theory affects social workers and provide evidence on how mindfulness practice may mitigate burnout amongst vulnerable social workers in China.

## Materials and Methods

### Data and Sample

The data for the study was collected through an online anonymous survey. The survey was distributed to selected front-line social workers in Guangzhou, China, which has seen rapid development in social work (Guangzhou Social Work Association, 2021). Since 2017, the Guangzhou government established street level social work service stations to provide accessible social services to the Guangzhou community. Essentially, the government purchases or contracts out social work services to registered social work agencies to provide the services at the workstations. Each station was equipped with 20 social workers, 14 of whom are front-line social workers. Of the 180 Guangzhou social work service stations, we randomly selected 54 stations to study.

On September 15, 2021, we sent the front-line workers a survey link to participate in the study. After this initial invitation, we sent the social workers two reminders to participate in the survey 7 and 14 days later. Out of 756 front-line social workers (54 × 14), 537 social workers participated in the online survey by October 10, 2021. The response rate was 71%. All participants went through an informed consent process, were notified of their ability to end the survey at will, and notified their participation was voluntary, prior to starting the survey. A research review committee at one of the co-author’s university in China approved this research method. A majority of the sample were female (84.5%) and never married (54.2%). The sample reported a mean age of 29-year-old. And more than half of the sample had at least a college degree, see Table 1.

**TABLE 1:** Descriptive statistics of key variables.

	Mean (S.D.)
1. Burnout (1–4)	2.5 (0.4)
2. Job demands (1–7)	4.7 (0.7)
3. Job resources (1–7)	5.2 (0.7)
4. Mindfulness practice (0–6)	2.8 (1.4)
Formal (0–6)	2.3 (1.8)
Informal (0–6)	3.3 (1.7)
5. Entertainment activities (0–6)	5.4 (1.3)
6. Female (%)	84.5
7. Age (18–60)	29.3 (6.3)
8. Education (%)	
Below college	45.8
College and above	54.2
9. Marital status (%)	
Never married	54.2
Married	45.8

*N = 537. Numbers in brackets show ranges of the variables.*

### Measures

Burnout was assessed by the Oldenburg Burnout Inventory (OBI; Demerouti and Bakker, 2008). The OBI’s psychometric soundness, reliability, and validity have been verified through samples of working professionals of numerous occupations, languages, and countries (Demerouti et al., 2001, 2003; Halbesleben and Demerouti, 2005). The survey consists of 16 items which measured the two-dimensional concept of burnout: exhaustion (8 items) and disengagement from work (8 items). Exhaustion is defined as a consequence of intense physical, affective, and cognitive strain. Disengagement refers to distancing oneself from one’s work in general, work object, and work content (Demerouti and Bakker, 2008). One of the example questions asked to assess burnout was: “It happens more and more often that I talk about my work in a negative way.”

The survey questions were written in English but were later translated to Chinese by two Chinese doctoral students in the United States. Additionally, an American professor whose native language is Chinese, verified these translations upon completion. Supplementary Appendix 1 lists the original version and translation of the scale, for each question. For both sub-scales, four items are positively worded, and four items are negatively worded. Each item was measured in four categories (1 = strongly disagree to 4 = strongly agree). We reversed positively worded items so that high scores represented high burnout. We averaged mean score of all 16 items as the burnout score. The Cronbach’s alpha of the scale was 0.85 in our study.

We used Lequeurre et al.’s (2013) Questionnaire sur les Ressources et Contraintes Professionnelles (QRCP) to measure JD-R. Considering Chinese social workers’ workload, we focused on three aspects of JD: pace and amount of workload, emotional workload, and changes in the tasks. In addition, we focused on three aspects of JR: relationships with colleagues, relationships with supervisors, and access to work-performance information or feedback. Pace and amount of workload refers to the experience of having excess work tasks within a limited time frame to accomplish them, while emotional workload encompasses the emotional energy employees are forced to expel to accomplish certain JD. And changes in the tasks refers to the difficulties posed to employees by changes in job roles and function. Relationships with colleagues refers to the teamwork environment and examines the levels of aid, support, and dependability co-workers provide one another at work. Relationships with supervisors describes how employees perceive their relationship with someone whose job role is above theirs. And access to work-performance information refers to the employee’s ability to receive feedback on his or her job performance.

Lequeurre et al. (2013) used 4 questions to measure each dimension. Example questions included “Do you have too much work to do?” and “Can you count on your colleagues when you encounter difficulties in your work?” (see Supplementary Appendix 1 for all questions). Every item was rated using a 7-point Likert scale ranging from 1 (never) to 7 (always). The higher the score per item, the higher the level of job demands, or job resources was present. We determined the scores of JD and JR by averaging the item responses under each scale. The Cronbach’s alpha was 0.83 for JD and 0.93 for JR.

Mindfulness practice was measured by asking respondents whether they participate in the following activities: meditation and mindful nature observation. We used meditation to gauge the extent of formal practice and utilized mindful nature observation to measure informal practice. Each activity was then rated on a 7-point Likert scale: 0 (never), 1 (once or less every 2 months), 2 (once per month), 3 (2 or 3 times per month), 4 (once per week), 5 (2 or 3 times per week), and 6 (once or more per day).

The analysis also considered certain demographics and socioeconomic characteristics from the participants, including gender (female = 1, male = 0), age, marital status (never married = 1, other = 0), and education (college degree or above = 1, below college education = 0). In addition, we included entertainment activities in the analysis to control for their effect on burnout. These entertainment activities included using social media, gaming, watching movies, etc., and were scored with the same rating system as MP.

### Analytical Approach

To begin, we implemented descriptive and correlation analyses to detect respondents’ characteristics and correlations among all variables. Second, we conducted an ordinary least squares (OLS) regression analysis to estimate the effects of JD and JR on burnout and whether the above associations were moderated by MP, while simultaneously controlling for socioeconomic characteristics of the respondents (Hayes, 2017). Specifically, we included all key variables as mentioned above in the first model. Next, we further examined the specific effects of formal or informal MP in the second and third models. In model 4, we tested the moderation effect by adding the interaction between JD and MP into the analysis. Alternatively, Structural Equation Modeling (SEM) can be used to test the moderation effects. We conducted a SEM analysis, and the results (available upon request) were no different from the regression approach. Ultimately, the regression approach was preferred as it allows us to take socioeconomic characteristics of the respondents and to conduct analyses on different MP specifications. All analyses used STATA software 16.0 to examine the data.

## Results

Table 1 demonstrates the variable’s descriptive statistics. Respondents reported a mean burnout score of 2.5, with a standard deviation of 0.4. Respondents conferred relatively high JD (*M* = 4.7, SD = 0.7) and JR (*M* = 5.2, SD = 0.7). This result suggests that despite experiencing high JD, respondents also had numerous JR such as support from coworkers or supervisors. The average MP was 2.8, ranging from 0–6. Broadly, social workers practiced informal MP around a couple of times per months (*M* = 3.3) and practiced formal MP about one time per month (*M* = 2.3). In contrast, social workers engaged in entertainment activities at a couple of times per week (*M* = 5.4).

The correlation analyses from Table 2, were consistent with our hypotheses. JD and burnout were positively correlated, (*r* = 0.51, *p* < 0.001) while JR and burnout were negatively correlated (*r* = –0.30, *p* < 0.001). MP, both formal and informal, were negatively correlated with burnout (*r* = −0.23, −0.11, −0.28, respectively). JR and MP had a positive correlation, (*r* = 0.15, *p* < 0.001) while there was no correlation between JD and MP. Formal and informal MP were also highly correlated with each other (*r* = 0.44, *p* < 0.001). Additionally, being of young age and having never married was positively correlated with burnout. There was also no substantial correlation between gender, education, and entertainment activities with burnout.

**TABLE 2:** Correlation analysis of key variables.

	1	2	3	4	5	6	7	8	9	10
1. Burnout	−									
2. Job demands	0.51***	−								
3. Job resources	−0.30***	0.04	−							
4. Mindfulness practice (MP)	−0.23***	–0.04	0.15***	−						
5. Formal MP	−0.11**	–0.01	0.08	0.86***	−					
6. Informal MP	−0.28***	–0.06	0.18***	0.83***	0.44***	−				
7. Entertainment activities	–0.02	–0.03	0.14**	0.07	–0.01	0.12**	−			
8. Female	–0.05	–0.03	–0.08	–0.06	–0.04	–0.06	−0.11*	−		
9. Age	−0.18***	–0.01	0.07	0.10*	0.02	0.16***	−0.09*	–0.01	−	
10. Collage and above education	0.07	0.08	–0.02	0.03	0.02	0.03	–0.00	–0.07	0.10*	−
11. Never married	0.18***	0.01	–0.01	−0.10*	–0.03	−0.14**	0.15***	−0.09*	−0.59***	–0.06

N = 537. *p < 0.05, **p < 0.01, ***p < 0.001.

Table 3 demonstrates the standardized estimates of burnout. Four models were presented. The first modeled MP included both formal and informal MP, while the second model focused on formal MP, and the third model focused on informal MP. The interaction between JD and MP were added in Model 4. The adjusted R-square of Model 1 was 0.41. As expected, JD and JR have significant effects on burnout. A one standard deviation increase in JD was associated with an increase of 0.51 standard deviations in burnout. An increase of one standard deviation in JR was associated with a decrease of 0.30 standard deviations in burnout. These results confirm Hypothesis 1. MP showed significant effects on reducing burnout (Beta = −0.15, *p* < 0.001), and the effects hold well for formal and informal MP in Model 2 (Beta = −0.08, *p* < 0.05) and 3 (Beta = −0.19, *p* < 0.001). These findings support Hypothesis 2.

**TABLE 3:** Regression analysis of burnout.

	Model 1			Model 2			Model 3			Model 4		
	Beta	S. E.	P	Beta	S. E.	P	Beta	S. E.	P	Beta	S. E.	P
Mindfulness practice	–0.15	0.01	***	−	−		−	−		0.57	0.05	**
Formal mindfulness practice	−	−		–0.08	0.01	*	−	−		−	−	
Informal mindfulness practice	−	−		−	−		–0.19	0.01	***	−	−	
Job demands	0.51	0.02	***	0.52	0.02	***	0.51	0.02	***	0.73	0.03	***
Mindfulness practice * Job demands	−	−		−	−		−	−		–0.76	0.01	***
Job resources	–0.30	0.02	***	–0.31	0.02	***	–0.29	0.02	***	–0.29	0.02	***
Entertainment activities	0.02	0.01		0.01	0.01		0.04	0.02		0.03	0.01	
Female	–0.06	0.03		–0.05	0.03		–0.06	0.03		–0.05	0.02	
Age	–0.07	0.01		–0.08	0.01		–0.06	0.00		–0.07	0.01	
Education – Collage and above	0.04	0.02		0.04	0.02		0.04	0.02		0.04	0.02	
Never married	0.11	0.03	**	0.12	0.03	**	0.10	0.03	*	0.12	0.03	**
Adjusted R-square	0.41			0.40			0.43			0.43		

N = 537. * p < 0.05, ** p < 0.01, *** p < 0.001.

**FIGURE 1:**
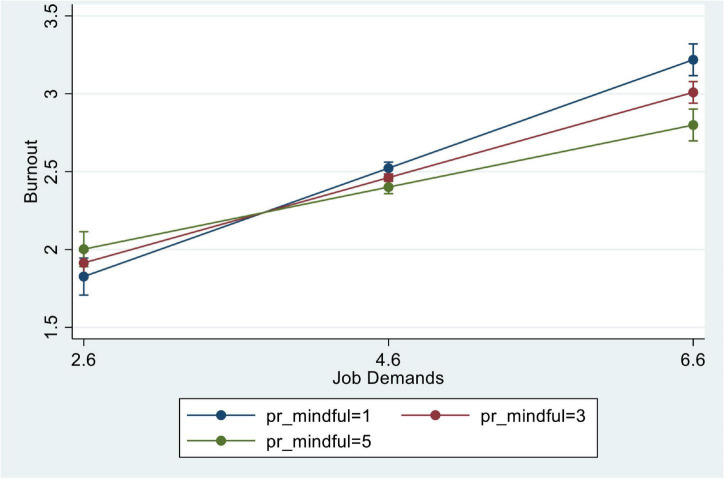
Job demands and burnout by mindful activities. The graph was based on results from Model 4 of Table 3.

The interaction between JD and MP showed significantly negative effects on burnout (Beta = −0.76, *p* < 0.001) in Model 4. The interaction effect, as shown in Figure 1, indicated that social workers with higher MP scores were less effected by burnout. The findings support Hypothesis 3.

## Discussion

The findings from the regression analysis supported the dual process hypothesis that JD-R affects burnout amongst Chinese social workers. The health-impairment process was indicated by the positive relationship between JD and burnout, whereas the motivation process was shown by negative association between JR and burnout. This means social workers are less likely to encounter burnout if they have high JR. However, the results also show that JD appear to have a greater effect on burnout amongst Chinese social workers than JR do. These results are compatible with what previous research has found in their application of the JD-R model (Demerouti et al., 2001; Bakker et al., 2003; Hakanen et al., 2008).

The regression results on MP, including both formal and informal ones, showed that MP had effects on reducing burnout of social workers in China. Previous studies have found MP reduces stress and increases mental health, which indicates the consistency of our results (Hanley et al., 2015; Yang et al., 2018; Birtwell et al., 2019; Shankland et al., 2020). The estimate of MP, however, suggests that the effects are likely to be small to medium. The finding that the estimate of informal MP was larger than the one found in formal MP was similar to the one found in Birtwell et al. (2019). However, further research is needed to comprehend the difference. Social workers may be more likely to take on informal MP because it requires less structure, is easier to perform, and derives the quickest benefit from the practice. This is especially likely as a recent study found that informal MP was associated with the observing facet of mindfulness (Cebolla et al., 2017). This ability to pay attention to experience is a key mindfulness skill that may reduce the extent of stress and burnout in daily work. On the other hand, people who engage in formal MP may be more aware of their stress and burnout at work, which might influence their self-reports to show higher levels of stress and burnout. Further research on formal and informal MP on burnout is warranted.

The findings of this study also have practice implications on the duty of social work employers. Given the average sample expressed high JD, and the positive relationship between JD and burnout, it is critical social work employers remain aware and cautious of projecting high JD onto their employees. In an effort to reduce burnout amongst employees, social work employers might consider creating supportive services and continue to maintain supportive work environments for their social workers. Because this may be challenging for smaller agencies with more limited resources to do, federal, provincial, and local policies should be redirected to help generate the funding and resources smaller agencies require to prevent burnout.

In addition, literature has shown a strong connection between burnout and mental illness. Thus, reducing burnout can also reduce mental illness (Kessler et al., 2010). The significant and negative interactive effect of JD and MP on burnout suggests that MP is likely to be a service or intervention that can be beneficial to social workers’ mental health who have high JD in China. This is particularly important for social workers in the sample who reported high JD, and also for the young and never married, who reported high burnout. Interestingly, social workers’ use of entertainment activities did not influence burnout, despite wide use amongst the sampled social workers. Entertainment activities may reduce stress temporarily but do not have a long-term effect on reducing burnout.

The study’s findings must also be evaluated with the context of several limitations. First, because the analysis was based on a cross-sectional dataset, the associative relationship between JD-R, MP, and burnout could only be approximated. Future studies could better approximate the causal relationships of these variables using a longitudinal design. Second, there may have been unobserved variables that were excluded from the study, and had effects on JD-R, MP, and burnout, such as job insecurity and suffering at work (Sánchez-Hernández et al., 2020; Stankevičiûtë et al., 2021). In addition, even though the sample focused on social workers generally, social workers can have a variety of rolls that could create undue influence on their response to JD-R, MP, and burnout. Individual participants may have varying personality traits (i.e., resilience) that also influence their experience with JD-R, MP, and burnout. Third, the data collected on JD-R, MP, and burnout were from the subjects’ self-reports which may have created intended and unintended reporting errors within the data. For example, subjects may have social desirability bias, which is the tendency to underreport socially undesirable attitudes and behaviors and to overreport more desirable attributes. Therefore, respondents may have underreported their JD while overreported their JR. Thus, future research should consider using other methods such as data triangulation through colleague and employer reports. Finally, the study’s results are based solely on social workers from one city, Guangzhou. Even though the sample size and response rate support confident results, it is unknown how generalizable the findings are to all Chinese social workers and thus require further investigation.

## Conclusion

This study utilized data from 537 social workers in Guangzhou, China, to better understand how the relationship between JD-R and MP affect burnout amongst Chinese social workers. Our findings were synonymous to past findings from cross-cultural research, which have indicated JD-R affects burnout. The results expand upon previous research and contribute to the JD-R theory by providing evidence on MP’s role on reducing burnout within a sample of Chinese social workers. The findings emphasize the significance of decreasing JD and increasing JR and MP for Chinese social workers, to protect workers and possibly limit the growing turnover rate.

## Data Availability Statement

The raw data supporting the conclusions of this article will be made available by the authors, without undue reservation.

## Ethics Statement

The studies involving human participants were reviewed and approved by Research Review Committee, School of Public Administration, Guangdong University of Foreign Studies. Written informed consent for participation was not required for this study in accordance with the national legislation and the institutional requirements.

## Author Contributions

BT, CH, SS, and MY: conceptualization, validation, and writing—original draft preparation. BT and CH: methodology and software, resources, and investigation and data curation. BT, MY, and CH: formal analysis. All authors contributed to the article and approved the submitted version.

## Conflict of Interest

The authors declare that the research was conducted in the absence of any commercial or financial relationships that could be construed as a potential conflict of interest.

## Publisher’s Note

All claims expressed in this article are solely those of the authors and do not necessarily represent those of their affiliated organizations, or those of the publisher, the editors and the reviewers. Any product that may be evaluated in this article, or claim that may be made by its manufacturer, is not guaranteed or endorsed by the publisher.
