# Supplementary material for: Mindfulness Practice and Burnout: Evidence From Chinese Social Workers
Source: Front Psychol. 2022 Feb 24;13:821899. doi: 10.3389/fpsyg.2022.821899 (PMC8907924; doi:10.3389/fpsyg.2022.821899)
Supplement: Supplementary file 1 [file Table_1.pdf]

Appendix 1: Original and Translation of the Scales

Original Version	Translation (Chinese)
**Scale: Burnout**	**量表：倦怠**
1.I always find new and interesting aspects in my work	1.我总是在工作中发现新的有趣的方面。
2.There are days when I feel tired before I arrive at work	2.有时候上班前我会感到疲倦。
3.It happens more and more often that I talk about my work in a negative way	3.我越来越多地以负面的方式谈论我的工作
4.After work, I tend to need more time than in the past in order to relax and feel better	4.下班后，我倾向于比过去需要更多的时间来放松和感觉更好。
5.I can tolerate the pressure of my work very well	5.我可以很好地承受工作的压力。
6.Lately, I tend to think less at work and do my job almost mechanically	6.最近，我倾向于在工作中少思考，几乎机械地做我的工作。
7.I find my work to be a positive challenge	7.我发现自己的工作是一个积极的挑战。
8.During my work, I often feel emotionally drained	8.在工作中，我经常会感到精神疲惫。
9.Over time, one can become disconnected from this type of work	9.随着时间的流逝，人们可能会与这种工作脱节。
10.After working, I have enough energy for my leisure activities	10.下班后，我有足够的精力进行休闲活动。
11.Sometimes I feel sickened by my work tasks	11.有时我对工作任务感到厌恶。
12.After my work, I usually feel worn out and weary	12.下班后，我通常会感到疲惫不堪。
13.This is the only type of work that I can imagine myself doing	13.这是我可以想象的唯一的工作类型。
14.Usually, I can manage the amount of my work well	14.通常，我可以很好地管理自己的工作量。
15.I feel more and more engaged in my work	15.我越来越多地喜欢我从事的工作。
16.When I work, I usually feel energized	16.工作时，我通常感到精力充沛。

Scale: Job Demands	量表：工作需求
1.Do you have too much work to do?	1.您有太多工作要做吗？
2.Do you have to work extra hard in order to complete something?	2.您是否需要付出更多努力才能完成某些工作？
3.Do you have to hurry?	3.您工作的节奏是否比较快?
4.Would you prefer a calmer work pace?	4.您是否希望工作节奏更平稳？
5.Does your work demand a lot from you emotionally?	5.您的工作对您的情感需求是否很高？
6.In your work, do you have to be able to convince or persuade people?	6.在工作中，您是否必须能够说服别人？
7.Are you confronted with things that affect you personally in your work?	7.您在工作中是否遇到会影响您个人的事情？
8.Does your work put you in emotionally upsetting situations?	8.您的工作是否使您陷入情绪低落的境地？
9.Have the proposed changes in your tasks been introduced well?	9.如果工作任务有改变，您是否得到了很好的说明?
10.Do you find it difficult to adapt to changes in your tasks?	10.您发现很难适应工作任务的改变吗？
11.Do the changes in your tasks cause you problems?	11.工作任务中的改变会造成您的困扰吗？
12.Do the changes in your tasks have negative consequences for you?	12.工作任务的改变对您有负面影响吗？
**Scale: Job Resources**	**量表：工作资源**
1.In your work, do you feel appreciated by your colleagues?	1.在工作中，您感到同事的赞赏吗？
2.Do you get on well with you colleagues?	2.你和同事相处得很好吗？
3.Can you count on your colleagues when you encounter difficulties in your work?	3.当您在工作中遇到困难时，您可以指望您的同事吗？
4.Is there a good atmosphere between you and your colleagues?	4.您和您的同事之间是否有良好的氛围？
5.In your work, do you feel appreciated by your superior?	5.在工作中，您是否感到上司的赞赏？
6.Do you get on well with you superior?	6.您和上司相处得好吗？
7.Can you count on your superior when	7.当您遇到工作上的困难时，您能指

you come across difficulties in your work?	望上司吗？
8.Is there a good atmosphere between you and your superior?	8.您和您的上级之间是否有良好的氛围？
9.Does your work give you the opportunity to check on how well you are doing your work?	9.您的工作是否使您有机会检查自己的工作状况？
10.Does your work provide you with direct feedback on how well you are doing your work?	10.您的工作是否为您提供有关工作进展情况的直接反馈？
11.Do you receive sufficient information on the results of your work?	11.您是否收到有关工作结果的足够信息？
12.Does your superior inform you about how well you are doing your work?	12.您的上司会告诉您，您的工作状况如何吗？
